# Newly Diagnosed Hepatic Encephalopathy Presenting as Non-convulsive Status Epilepticus: A Case Report and Literature Review

**DOI:** 10.3389/fneur.2022.880068

**Published:** 2022-05-12

**Authors:** Marco Olivero, Delia Gagliardi, Gianluca Costamagna, Daniele Velardo, Francesca Magri, Fabio Triulzi, Giorgio Conte, Giacomo P. Comi, Stefania Corti, Megi Meneri

**Affiliations:** ^1^Neuroscience Section, Dino Ferrari Centre, Department of Pathophysiology and Transplantation (DEPT), University of Milan, Milan, Italy; ^2^Neurology Unit, Foundation IRCCS Ca' Granda Ospedale Maggiore Policlinico, Milan, Italy; ^3^Neuromuscular and Rare Diseases Unit, Department of Neuroscience, Fondazione IRCCS Ca' Granda Ospedale Maggiore Policlinico, Milan, Italy; ^4^Neuroradiology Unit, Fondazione IRCCS Ca' Granda Ospedale Maggiore Policlinico, Department of Pathophysiology and Transplantation, Università degli Studi Milano, Milan, Italy

**Keywords:** hepatic encephalopathy, non-convulsive status epilepticus, brain magnetic resonance imaging, case report, corticospinal tract, globus pallidus

## Abstract

**Background:**

Hepatic encephalopathy is characterized by psychiatric and neurological abnormalities, including epileptic seizure and non-convulsive and convulsive status epilepticus. Conventional brain magnetic resonance imaging is useful in supporting diagnosis since it can reveal specific radiological findings. In the literature, there is no description of hepatic encephalopathy onset as non-convulsive status epilepticus; we provide the first report.

**Case Summary:**

We report a case of a 67-year-old woman, without history of cirrhosis, presenting altered mental state, normal brain computed tomography imaging, and electroencephalography suggestive of epileptic activity. We suspected non-convulsive status epilepticus, and we administered diazepam and levetiracetam with clinical improvement. Thus, we made a diagnosis of non-convulsive status epilepticus. A radiological study with brain magnetic resonance imaging showed bilateral hyperintensity on T1-weighted sequences of globus pallidus and hyperintensity of both corticospinal tracts on T2-weighted fluid-attenuated inversion recovery sequences. Blood tests revealed hyperammonemia, mild abnormality of liver function indices, and chronic Hepatitis B and D virus coinfection. Hepatic elastosonography suggested liver cirrhosis. The patient started antiviral therapy with entecavir and prevention of hepatic encephalopathy with rifaximin and lactulose; she was discharged with a normal mental state.

**Conclusions:**

Hepatic encephalopathy can present as an initial manifestation with non-convulsive status epilepticus. Electroencephalography is useful for differentiating non-convulsive status epilepticus from an episode of hepatic encephalopathy, and neuroimaging aids the diagnostic process.

## Introduction

Hepatic encephalopathy (HE) is defined as brain dysfunction caused by liver failure or portal systemic shunting, without considering the etiology (1). The clinical picture comprises neurological or psychiatric abnormalities, ranging from subclinical alterations to coma (1). It is one of the most important complications of liver cirrhosis, contributing to both morbidity and mortality (1). From the pathophysiological perspective, ammonia is, probably, the central player in the pathogenesis of HE (1). Considered a metabolic disorder, it is usually reversible by liver transplantation (1). Although rare, HE can present as epileptic seizure; manifestations vary widely, encompassing tonic-clonic seizure, convulsive (CSE), and non-convulsive status epilepticus (NCSE) (2–6). HE can show typical features on brain magnetic resonance imaging (MRI), namely, the bilateral and symmetric hyperintensity of the globus pallidus on T1-weighted (T1W) sequences, and hyperintensity in cerebral white matter, involving corticospinal tract and subcortical hemispheric white matter on T2 weighted (T2W)—“fluid attenuated inversion recovery” (FLAIR) MRI sequences (7). Herein, we report a case of newly diagnosed HE presented as NCSE, in which conventional brain MRI shows some findings associated with this disease, such as the hyperintensity in the globus pallidus on T1 and the hyperintensity along the corticospinal tract on T2-FLAIR.

## Case Presentation

**Figure 1:**
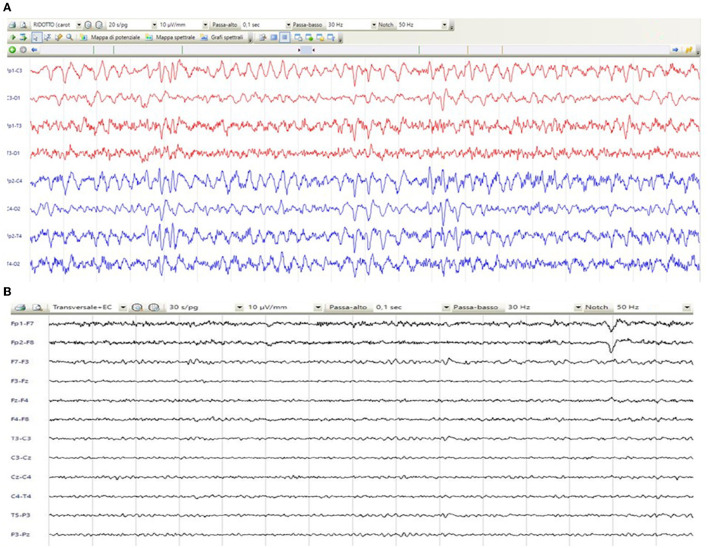
EEG showing status epilepticus **(A)** and resolution after antiepileptic therapy **(B)**.

**Figure 2:**
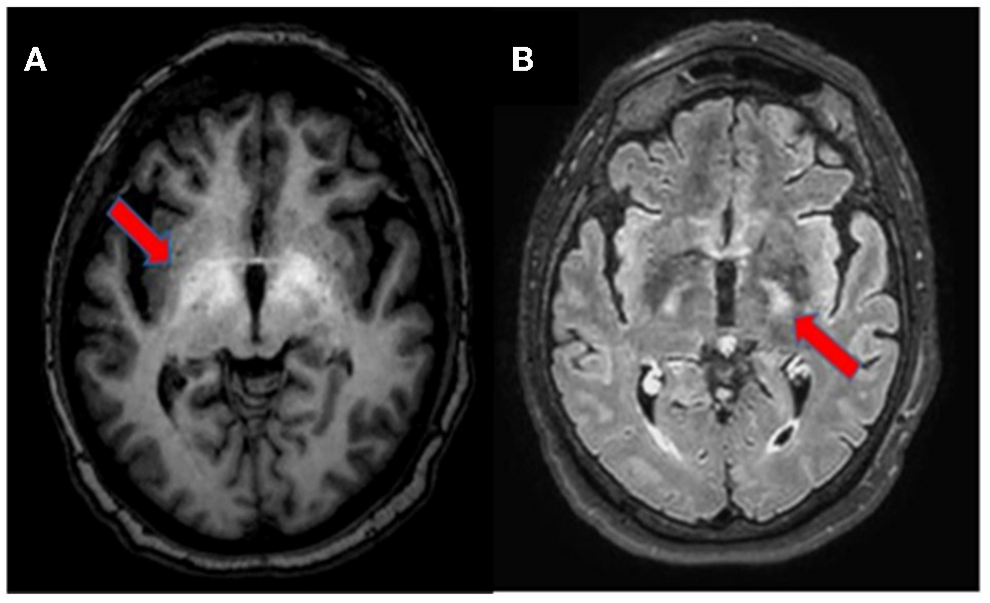
Brain MRI, axial section. T1-weighted imaging showing bilateral symmetrical hyperintensity in the globus pallidus and upper mesencephalon [**(A)**, arrow]. T2-weighted FLAIR imaging depicting hyperintensity of the corticospinal tracts [**(B)**, arrow].

**Figure 3:**
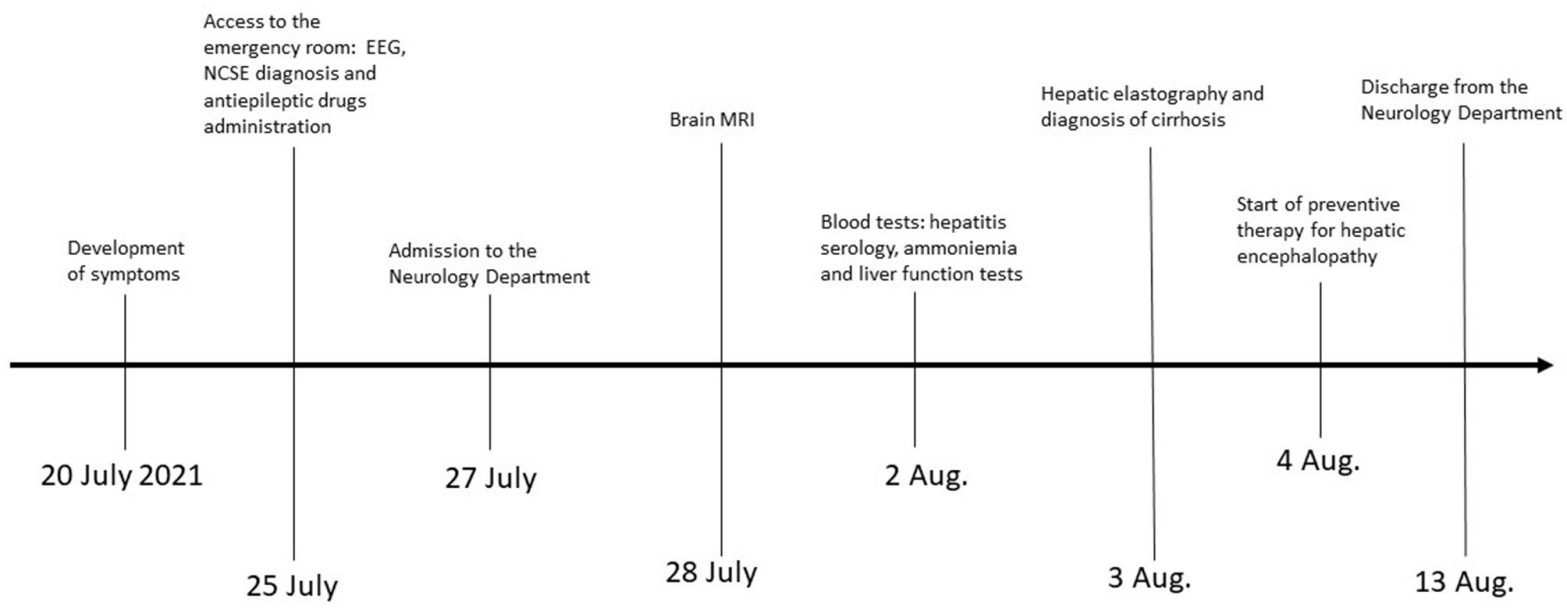
The timeline of the case report.

A 67-year-old woman from Romania presented to our Emergency Department for confusion and subacute ideomotor decline in the previous 5 days. Insomnia, nocturnal awakenings, urinary incontinence, and amnesia for recent events were reported in the last month. The patient had a past medical history of paroxysmal atrial fibrillation, previously treated with amiodarone and apixaban, which were self-suspended 1 year ago, obesity, hypertension, and rectal carcinoma which was surgically treated with an enterostomy. The patient had no known history of seizures or other neurological diseases, recent illness, brain trauma, or recent surgical procedures. The chronic therapy encompassed olmesartan, bisoprolol, indapamide, furosemide, amlodipine, acetylsalicylic acid, and ranitidine. The vital parameters were normal, and the Glasgow Coma Scale (GCS) score was 15. The general physical examination did not show pathological items, nor signs of trauma. The chest X-ray was normal, while the electrocardiogram was suggestive of atrial fibrillation. From a neurological point of view, the patient was disoriented in space and time and unable to denominate objects of common use. The pupils were isochoric and reactive to light. The posture was normal and there were no signs of meningism. The cranial nerve examination was normal, except for dubious absence of the menace reflex on the left, not confirmed by a subsequent evaluation. The global strength of the limbs was preserved, as well as tactile sensitivity and coordination. The osteotendinous reflexes were valid, symmetrical in the upper limbs, with a mild prevalence on the right, and weak and symmetrical in the lower limbs. Cutaneous plantar response was mute bilaterally. A stroke was initially suspected, and a neuroimaging study was performed with brain computed tomography (CT), CT angiography, and CT perfusion, which were unremarkable. Because of the persistence of the altered mental state, electroencephalography (EEG) was performed. EEG showed a broadly slowed trace, with a background theta rhythm, which was more expressed on the right. Fast paroxysmal bilateral activity of the type sharp wave was superimposed and more represented on the right and mainly frontotemporal (Figure 1A) areas. Herein, NCSE was suspected and 10 mg of diazepam was administered intravenously, with regression of the paroxysmal activity on the synchronous recording of the EEG. Furthermore, antiepileptic therapy with 4,000 mg of intravenous levetiracetam was started, and the mental state progressively improved. The next day, the EEG was repeated, and epileptiform activity did not reappear (Figure 1B); levetiracetam was switched to an oral dosage of 2,000 mg daily. The patient was admitted to our Neurology Department with the presumptive diagnosis of NCSE, and a 3 Tesla brain MRI with gadolinium was performed. Bilateral hyperintensity of the lenticular nucleus was detected on T1W sequences (Figure 2A), while bilateral hyperintensity of the corticospinal tract signal was displayed on FLAIR (Figure 2B). Neuroimaging ruled out an ischemic event and an expansive lesion, with findings suggestive of chronic HE. As a completion, a lumbar puncture with examination of cerebral spinal fluid was performed. It did not show any noteworthy findings, including the real-time polymerase chain reaction for an infectious agent or dosage of autoantibodies associated with autoimmune and paraneoplastic encephalitis. The patient was obese (body mass index = 38.3 kg/m^2^) but had no past history of alcoholic abuse. There were no signs of ascites and declining edema. She presented no signs of asterixis. Blood tests performed a few days later revealed: chronic coinfection of Hepatitis B (HBV) and D (HDV) virus, negative Hepatitis C virus (HCV) infection, ammonium 141 umol/L (11.2–48.2 umol/L), albumin 2.7 mg/dl (3.4–4.8), total bilirubin 1.33 (0.12–1.1 mg/dl), INR 1.25 (0.8–1.2), proteins 5.6 g/dl (6.4–8 g/dl), cobalamin 1,121 pg/ml (191–663 pg/ml), and normal levels of folate, transferrin, sideremia, and ferritin. Hepatic elastography recorded values compatible with cirrhosis. Hepatic cirrhosis from mixed etiology, chronic HBV-HDV co-infection, and metabolic syndrome was diagnosed. The clinical picture was considered to be an episode of HE, presenting as NCSE. Anti-HBV therapy with entecavir (0.5 mg daily orally) was started, as well as secondary prevention of HE with lactulose (10 g two times daily orally) and rifaximin (400 mg three times daily orally). On the day of discharge, her mental state was normal, and the patient noted improvement in her general health condition (Figure 3). Afterwards, she continued to receive levetiracetam for epilepsy, besides lactulose and rifaximine for secondary prevention of HE; relatives reported no other episodes of mental confusion. Three months later, a brain MRI was repeated, and radiological findings were stable.

## Discussion

HE is one of the main complications of liver cirrhosis, along with variceal bleeding, hepatorenal syndrome, hepatopulmonary syndrome, and ascites (8). Historically, it has been classified into “overt hepatic encephalopathy” (OHE, clinically manifested neurological and psychiatric abnormalities) and “covert hepatic encephalopathy” (CHE, abnormalities on neuro-psychological and electrophysiological tests without or mild clinically detectable neurological-psychiatric abnormalities) (9). According to the recent “International Society for Hepatic Encephalopathy and Nitrogen Metabolism” (ISHEN) consensus, the onset of disorientation and/or asterixis confirms OHE (10). In cirrhotic patients, OHE develops in 30–40% at some time during their clinical course, while minimal hepatic encephalopathy (MHE) and CHE are seen in 20–80% (11). As HE is a manifestation of serious liver impairment, its outcome depends on the severity of underlying liver disease, its clinical course, and its treatment (1). The serum level of ammonia plays a central role in the pathophysiology of HE, helping physicians in ruling out the diagnosis and defining the prognosis; lowering serum ammonia is the main therapeutic goal (1, 9). Apart from hyperammonemia, other pathogenetic factors are implied, such as systemic inflammation, increased blood manganese, circulating bile acids, and lactate (1, 9). Their generation is influenced by common precipitant factors of HE, like malnutrition, infections, electrolyte imbalance, constipation, gastrointestinal bleeding, dehydration, and use of diuretics (1, 9). These pathogenetic factors influence the blood-brain barrier (BBB) by increasing its permeability (1, 9). Anyway, independent of blood-brain barrier status, ammonia passes freely into the brain, which is exclusively removed by astrocytes *via* glutamine synthetase (1, 9). The generation of glutamine renders the astrocyte hypertonic, resulting in swelling, impaired function, and, finally, brain edema (1, 9). Astrocyte swelling causes neuronal dysfunction and clinical manifestations of HE (1, 9). However, as in our case, there may be no clear precipitant factor underlying an episode of OHE (1, 9).

On a neurological point of view, clinical elements are plethoric, encompassing, mostly, the higher cortical functions and the motor system (12). Cognitive findings in patients with chronic HE vary from subtle deficits, not apparent without psychometric and electrophysiological testing (CHE), to clearer findings during periods of decompensation related to higher ammonia levels, such as impairments in attention span, reaction time, and working memory (OHE) (12). Disturbances in the sleep-wake pattern are common initial manifestations of HE and may precede mental state changes or neuromuscular symptoms (13). As HE progresses, patients may develop mood and personality changes, disorientation in time and space, inappropriate behavior, somnolence, confusion, and, finally, the so-called “coma hepaticus” (14). Seizures and status epilepticus are very rarely reported in HE (2). Generally, HE is associated with different EEG patterns, such as delta activity and the more typical triphasic waves (9). In particular, the description of NCSE in HE is anecdotal, probably because cognitive disturbances are shared in the two conditions, and an EEG is not always performed. To the best of our knowledge, just four case reports were previously described, and none of them reported NCSE as the first manifestation of HE (3–6). In our case, NCSE was diagnosed according to Salzburg criteria, since we found epileptiform discharge on the EEG that ceased after antiepileptic drugs administration, associated with a mental state improvement (15). The pathophysiology underlying the development of seizures in the setting of HE remains unknown, although hyperammonemia is likely the most important factor (16). In our case, the patient developed, within a few days, cognitive symptoms of episodical OHE but more likely caused by NCSE secondary to hyperammonemia. Neuroimaging excluded structural causes of seizures, while blood tests showed no other noteworthy metabolic alterations.

Brain MRI is the most useful imaging technique to support the diagnosis of HE in uncertain cases (17). Neuroradiological signs depend on the severity and velocity of development of liver failure, namely, a chronic or acute HE (18). In this case, we observed findings on conventional brain MRI compatible with a chronic form of HE, probably long neglected, not associated with any neurological signs. The bilateral and symmetric T1 hyperintensity involving the globus pallidus and substantia nigra reticulata is characteristic, reported in almost 90% of patients with cirrhosis and likely due to manganese accumulation; it may be associated with Parkinsonism (7, 17). Sometimes, other areas are involved, namely, the subthalamic nucleus, tectal plate, hypothalamus, adenohypophysis, limbic system, and white matter (7, 17, 18). Less commonly, the hyperintensity of both corticospinal tracts is described on T2-FLAIR sequences; it is caused by vasogenic brain edema due to glutamine increase in astrocytes, resulting in loss of organic osmolytes, such as myo-inositol, that accumulate in the extracellular compartment (17). This finding may be associated with subclinical alterations, detectable in electrophysiological studies with motor-evoked potentials, not performed in the case of our patient (19). Similar abnormalities may also be seen in periventricular white matter, the thalamus, posterior limb of internal capsule, and cortex (18). However, hyperintensity along both the corticospinal tracts may be seen in healthy adults and should be interpreted with caution, especially in 3 Tesla MRI (17, 20). These changes are similar to the signal abnormalities observed in patients with other diseases, such as amyotrophic lateral sclerosis, X-linked Charcot-Marie-Tooth, optic neuromyelitis, metabolic disorders (Krabbe disease and X-linked adrenoleukodystrophy), infectious diseases (*Borrelia* spp. and Human T-cell lymphotropic Virus 1), and primary central nervous system lymphoma (21–28). Other neuroradiological findings include an increase of mean diffusivity of hemispheric brain matter on diffusion weighted imaging (DWI); laminar hyperintensities involving the cortical deep layers; white matter focal lesions; and a low magnetization transfer ratio (MTR) in various white matter regions (17, 29). These radiological findings have been shown to be reversible after liver transplantation (30, 31).

## Conclusions

We reported the first description of NCSE as an initial manifestation of HE. Specific brain MRI findings, namely, bilateral hyperintensity on T1 of the globus pallidus and hyperintensity of both corticospinal tracts on T2-FLAIR, were able to suggest the diagnosis of HE. From a general point of view, NCSE is easily underdiagnosed for lack of clear and univocal clinical signs. It is important to understand the possibility of NCSE in patients presenting an altered mental state; EEG is helpful in ruling it out. Hyperammonemia is a possible pathogenetic factor of NCSE, especially in patients with cirrhosis. The problem arises in differentiating acute alteration of the mental state due to an episode of HE from a real NCSE. Again, the EEG is useful for resolving diagnostic doubt, especially in the case of presumed HE that does not respond to empirical treatment.

## Data Availability Statement

The original contributions presented in the study are included in the article/supplementary material, further inquiries can be directed to the corresponding author/s.

## Ethics Statement

Written informed consent was obtained from the individual(s) for the publication of any potentially identifiable images or data included in this article.

## Author Contributions

MO: drafted the manuscript for intellectual content and collected and analyzed the data. DG, GCos, DV, and MM: collected and analyzed the data and revised the manuscript for intellectual content. FM, FT, GCon, GPC, and SC: revised the manuscript for intellectual content. All authors contributed to the article and approved the submitted version.

## Conflict of Interest

The authors declare that the research was conducted in the absence of any commercial or financial relationships that could be construed as a potential conflict of interest.

## Publisher's Note

All claims expressed in this article are solely those of the authors and do not necessarily represent those of their affiliated organizations, or those of the publisher, the editors and the reviewers. Any product that may be evaluated in this article, or claim that may be made by its manufacturer, is not guaranteed or endorsed by the publisher.

## References

[1] Rose CF Amodio P Bajaj JS Dhiman RK Montagnese S Taylor-Robinson SD et al. Hepatic encephalopathy: novel insights into classification, pathophysiology and therapy. J Hepatol. (2020) 73:1526–47. 10.1016/j.jhep.2020.07.013 33097308

[2] Rudler M Marois C Weiss N Thabut D Navarro V. Brain-Liver Pitié-Salpêtrière Study Group (BLIPS). Status epilepticus in patients with cirrhosis: How to avoid misdiagnosis in patients with hepatic encephalopathy. Seizure. (2017) 45:192–7. 10.1016/j.seizure.2016.12.011 28092846

[3] Jhun P Kim H. Nonconvulsive status epilepticus in hepatic encephalopathy. West J Emerg Med. (2011) 12:372–4. 10.5811/westjem.2011.1.2125 22224122 PMC3236148

[4] Badshah MB Riaz H Aslam S Badshah MB Korsten MA Munir MB. Complex partial nonconvulsive status epilepticus masquerading as hepatic encephalopathy: a case report. J Med Case Rep. (2012) 6:422. 10.1186/1752-1947-6-422 23244300 PMC3560269

[5] Jo YM Lee SW Han SY Baek YH Ahn JH Choi WJ et al. Nonconvulsive status epilepticus disguising as hepatic encephalopathy. World J Gastroenterol. (2015) 21:5105–9. 10.3748/wjg 25945028 PMC4408487

[6] Hor S Chen CY Tsai ST. Propofol pump controls nonconvulsive status epilepticus in a hepatic encephalopathy patient: a case report. World J Clin Cases. (2019) 7:2831–7. 10.12998/wjcc.v7 31616699 PMC6789384

[7] Rovira A Alonso J Córdoba J MR. imaging findings in hepatic encephalopathy. AJNR Am J Neuroradiol. (2008) 29:1612–21. 10.3174/ajnr.A1139 18583413 PMC8118773

[8] Tsochatzis EA Bosch J Burroughs AK. Liver cirrhosis. Lancet. (2014) 383:1749–61. 10.1016/S0140-6736(14)60121-5 24480518

[9] Wijdicks EF. Hepatic encephalopathy. N Engl J Med. (2016) 375:1660–70. 10.1056/NEJMra1600561 27783916

[10] Bajaj JS Wade JB Sanyal AJ. Spectrum of neurocognitive impairment in cirrhosis: Implications for the assessment of hepatic encephalopathy. Hepatology. (2009) 50:2014–21. 10.1002/hep.23216 19787808

[11] Vilstrup H Amodio P Bajaj J Cordoba J Ferenci P Mullen KD et al. Hepatic encephalopathy in chronic liver disease: 2014 Practice Guideline by the American Association for the Study of Liver Diseases and the European Association for the Study of the Liver. Hepatology. (2014) 60:715–35. 10.1002/hep.27210 25042402

[12] Dellatore P Cheung M Mahpour NY Tawadros A Rustgi VK. Clinical manifestations of hepatic encephalopathy. Clin Liver Dis. (2020) 24:189–96. 10.1016/j.cld.2020.01.010 32245526

[13] Córdoba J Cabrera J Lataif L Penev P Zee P Blei AT. High prevalence of sleep disturbance in cirrhosis. Hepatology. (1998) 27:339–45. 10.1002/hep.510270204 9462628

[14] Weissenborn K. Diagnosis of encephalopathy. Digestion. (1998) 59 Suppl 2:22–4. 10.1159/000051415 9718414

[15] Leitinger M Beniczky S Rohracher A Gardella E Kalss G Qerama E et al. Salzburg consensus criteria for non-convulsive status epilepticus—approach to clinical application. Epilepsy Behav. (2015) 49:158–63. 10.1016/j.yebeh.2015.05.007 26092326

[16] Ficker DM Westmoreland BF Sharbrough FW. Epileptiform abnormalities in hepatic encephalopathy. J Clin Neurophysiol. (1997) 14:230–4. 10.1097/00004691-19970500000008 9244163

[17] Alonso J Córdoba J Rovira A. Brain magnetic resonance in hepatic encephalopathy. Semin Ultrasound CT MR. (2014) 35:136–52. 10.1053/j.sult.2013.09.008 24745889

[18] Bathla G Hegde AN MRI. and CT appearances in metabolic encephalopathies due to systemic diseases in adults. Clin Radiol. (2013) 68:545–54. 10.1016/j.crad.2012.05.021 23142023

[19] Córdoba J Raguer N Flavià M Vargas V Jacas C Alonso J et al. T2 hyperintensity along the cortico-spinal tract in cirrhosis relates to functional abnormalities. Hepatology. (2003) 38:1026–33. 10.1053/jhep.2003.50406 14512890

[20] Neema M Guss ZD Stankiewicz JM Arora A Healy BC Bakshi R. Normal findings on brain fluid-attenuated inversion recovery MR images at 3T. AJNR Am J Neuroradiol. (2009) 30:911–6. 10.3174/ajnr.A1514 19369605 PMC3003332

[21] Kono Y Sengoku R Mitsumura H Bono K Sakuta K Yamasaki M et al. Clinical characteristics associated with corticospinal tract hyperintensity on magnetic resonance imaging in patients with amyotrophic lateral sclerosis. ClinNeurolNeurosurg. (2014) 127:1–4. 10.1016/j.clineuro.2014.09.011 25306412

[22] Kassubek J Bretschneider V Sperfeld AD. Corticospinal tract MRI hyperintensity in X-linked Charcot-Marie-Tooth Disease. J ClinNeurosci. (2005) 12:588–9. 10.1016/j.jocn.2004.07.020 16051098

[23] Zhu R Liu X He Z. Widely spread corticospinal tracts lesions in a case of neuromyelitisoptica. Clin Neurol Neurosurg. (2017) 161:56–8. 10.1016/j.clineuro.2017.08.010 28858632

[24] Demaerel P Wilms G Verdru P Carton H Baert AL MR. findings in globoid cell leucodystrophy. Neuroradiology. (1990) 32:520–2. 10.1007/BF02426470 2287386

[25] Loes DJ Fatemi A Melhem ER Gupte N Bezman L Moser HW et al. Analysis of MRI patterns aids prediction of progression in X-linked adrenoleukodystrophy. Neurology. (2003) 61:369–74. 10.1212/01.wnl.0000079050.91337.83 12913200

[26] Pruvost-Robieux E Yeung J Sudacevschi V Cordoliani Y De Malherbe M Pico F. Reversible corticospinal tract hyperintensities in neurologic Lyme disease. Neurology. (2016) 87:548–9. 10.1212/WNL.0000000000002913 27481458

[27] Konagaya M Iida M. A case of HTLV-1 associated myelopathy with diffuse white matter lesion of the frontal lobe and continuous lesion of the pyramidal tract on cranial MRI. Rinsho Shinkeigaku. (1991) 31:875–7. 1764864

[28] Shi K Shen J Yue X. Primary central nervous system lymphoma with symmetrical pyramidal tract hyperintensity. JAMA Neurol. (2021) 78:876–7. 10.1001/jamaneurol.2021.1165 33999112

[29] Matsusue E Kinoshita T Ohama E Ogawa T. Cerebral cortical and white matter lesions in chronic hepatic encephalopathy: MR-pathologic correlations. AJNR Am J Neuroradiol. (2005) 26:347–51. 15709133 PMC7974098

[30] Pujol A Pujol J Graus F Rimola A Peri J Mercader JM. Hyperintenseglobus pallidus on T1-weighted MRI in cirrhotic patients is associated with severity of liver failure. Neurology. (1993) 43:65–9. 10.1212/wnl.43.1_part_1.65 8423913

[31] Rovira A Córdoba J Sanpedro F Grivé E Rovira-Gols A Alonso J. Normalization of T2 signal abnormalities in hemispheric white matter with liver transplant. Neurology. (2002) 59:335–41. 10.1212/wnl.59.3.335 12177365

